# Prognostic nutritional index as a predictor of cardiovascular and all-cause mortality in American adults with hypertension: results from the NHANES database

**DOI:** 10.3389/fcvm.2024.1465379

**Published:** 2025-01-06

**Authors:** Jing Tang, Long Yang, Guan-Ying Yang, Yan-Hong Li, You-Sen Zhu, Hui Li, Xiao-Ming Gao

**Affiliations:** ^1^ State Key Laboratory of Pathogenesis, Prevention and Treatment of High Incidence Diseases in Central Asia, Department of Cardiology, First Affiliated Hospital of Xinjiang Medical University, Urumqi, China; ^2^ Clinical Laboratory, First Affiliated Hospital of Xinjiang Medical University, Urumqi, China; ^3^ Pediatric Cardiothoracic Surgery, First Affiliated Hospital of Xinjiang Medical University, Urumqi, China; ^4^ Pharmacy Department, Fifth Affiliated Hospital of Xinjiang Medical University, Urumqi, China; ^5^ Xinjiang Key Laboratory of Medical Animal Model Research, Xinjiang Medical University, Urumqi, China; ^6^ Clinical Medical Research Institute, Xinjiang Medical University, Urumqi, China

**Keywords:** prognostic nutritional index, hypertension, all-cause mortality, cardiovascular mortality, cohort study

## Abstract

**Background:**

Few studies have examined the relationship between nutritional status, as assessed by the Prognostic Nutrition Index (PNI), and incident cardiovascular mortality and all-cause mortality, particularly in hypertensive patients. This study aimed to examine the association between PNI and cardiovascular mortality and all-cause mortality in Americans with hypertension.

**Methods:**

Data from this retrospective cohort study were obtained from the National Health and Nutrition Examination (NHANES) 1999–2016. Using data of The NHANES Public-Use Linked Mortality Files to assess all-cause mortality (ACM) and cardiovascular mortality (CVM). After excluding participants younger than 18 years, without hypertension, and with missing follow-up data, a total of 18,189 cases were included in this study. Persons with hypertension were divided by PNI into 4 groups: Q1 (PNI < 49.0), Q2 (PNI: 49.0–52.5), Q3 (PNI: 52.5–55.5), and Q4 (PNI > 55.5). We used the Cox proportional hazard regression model to explore the predictive role of PNI on ACM and CVM in American adults with hypertension. Restricted cubic spline (RCS) curves to investigate the existence of a dose-response linear relationship between them.

**Result:**

During a median follow-up period of 89 months, a total of 1,444 (7.94%) cardiovascular deaths occurred and 5,171 (28.43%) all-cause deaths occurred. Multifactorial COX regression analysis showed all-cause mortality [hazard ratio (HR): 0.584, 95% CI: 0.523–0.652, *p* < 0.001] and cardiovascular mortality (HR: 0.435, 95% CI: 0.349–0.541, *p* < 0.001) associated with Q4 group risk of malnutrition in PNI compared to Q1 group. RCS curves showed a nonlinear relationship between PNI and all-cause mortality and cardiovascular mortality (both non-linear *p* < 0.001).

**Conclusions:**

Lower PNI levels are associated with mortality in patients with hypertension. PNI may be a predictor of all-cause mortality and cardiovascular mortality risk in patients with hypertension.

## 1 Introduction

As a common chronic disease, hypertension is a major public health problem and the greatest attributable risk factor for death worldwide (1). It is also a major modifiable risk factor for cardiovascular disease (CVD) and accounts for approximately 45% of global CVD morbidity and mortality (2, 3). Recently, there has been a sustained increase in the prevalence of hypertension globally. The current prevalence of hypertension is approximately 25% among adults and is expected to reach 29% by 2025, equating to approximately 1.55 billion people (4, 5).

Essential hypertension (EHTN) is the most common type of hypertension, accounting for 90%–95% of all cases. EHTN does not have a clear etiology but results from the combined effects of multiple factors (6). Its causes involve genetic susceptibility and environmental influences, while its pathophysiological mechanisms are associated with dysregulation of systems such as the renin-angiotensin-aldosterone system (RAAS), sympathetic nervous activity, and hormonal secretion (7, 8). Furthermore, research has indicated that inflammation is also a critical mechanism in hypertension, characterized by elevated levels of pro-inflammatory cytokines. In many chronic systemic diseases, impaired nutritional status is linked to increased morbidity and mortality (9). Numerous studies have indicated that immunological and nutritional statuses are closely related to cardiovascular progression and prognosis (10, 11). Body mass index (BMI), serum albumin (ALB) level, and pre-albumin (PA) levels are the most commonly used indexes to clinically evaluate nutritional status (12). However, these single indexes have limited clinical application. In recent years, research on the prognostic nutritional index (PNI) has become extremely popular. It was reported that PNI, calculated by serum albumin levels and peripheral lymphocyte, could reflect the nutritional and immunological status (13).

PNI was widely examined in patients with malignancy and in those undergoing surgery (14). Recently, this novel, composite, and objective marker of immunonutritional status indicator was shown to predict mortality in patients with ST elevated myocardial infarction (STEMI), acute heart failure (HF), and stable angina pectoris (SAP) (15, 16). However, the relationship between immunonutritional status and hypertensive cardiac disease has not been studied yet. Therefore, this study aimed to examine the association between PNI and cardiovascular mortality and all-cause mortality in Americans with hypertension.

## 2 Methods

### 2.1 Study population

**Figure 1:**
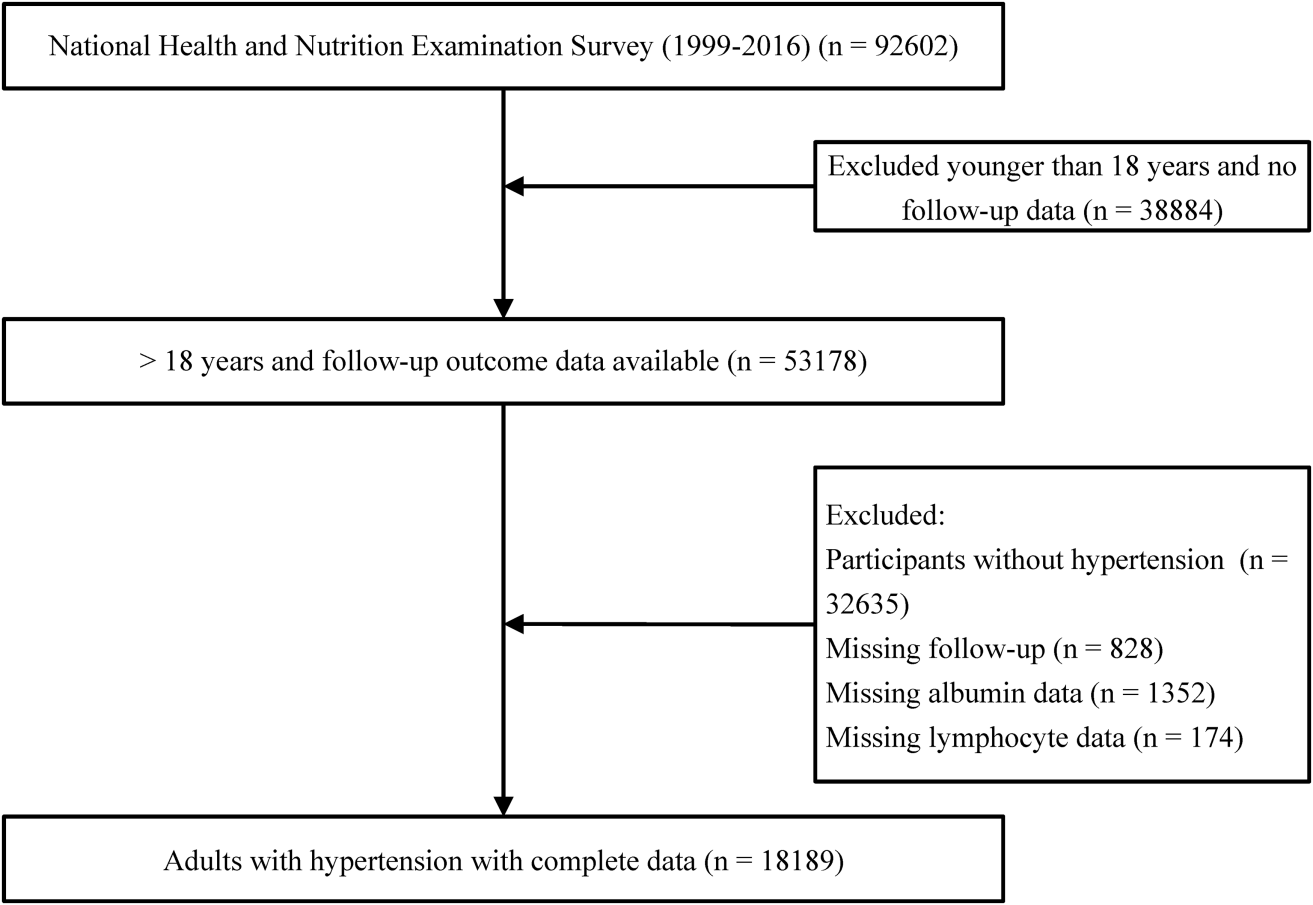
Flow diagram of patient selection.

This is a retrospective cohort study that utilized data from the National Health and Nutrition Examination Survey (NHANES). NHANES gathers information from participants every two years. The dataset employed in this study encompasses data from nine NHANES cycles spanning from 1999 to 2016. For this investigation, individuals under the age of 18 were excluded (*n* = 38,884), as were participants without hypertension (*n* = 32,635). Furthermore, cases with missing follow-up data (*n* = 828), serum albumin (*n* = 1,352), and lymphocytes (*n* = 174) were excluded, resulting in a final sample of 18,189 individuals (Figure 1). This research received support from the National Center for Health Statistics Research Ethics Review Board, and all participants provided written informed consent.

### 2.2 Diagnostic criteria for hypertension

First, information regarding hypertension history and the use of antihypertensive medications was collected using a structured questionnaire. Additionally, trained physicians carefully recorded blood pressure readings from the upper arm using a mercury sphygmomanometer. Each participant's blood pressure was measured three consecutive times after resting quietly in a seated position for at least 5 min. The average of these three readings was calculated as the participant's blood pressure. Hypertension was diagnosed if any of the following criteria were met (17): an average systolic blood pressure (SBP) ≥ 140 mmHg, an average diastolic blood pressure (DBP) ≥ 90 mmHg, self-reported history of hypertension, or previous use of antihypertensive medications.

### 2.3 Nutritional status assessment

In this study, the nutritional status of hypertensive participants was primarily assessed using the PNI. The PNI was calculated using the following formula: serum albumin (g/L) + 5 × total lymphocyte count (10^9 ^/L) (18). Additionally, the Geriatric Nutritional Risk Index (GNRI) (19) and Controlling Nutritional Status (CONUT) (20) were separately calculated to compare the results with other scores.

### 2.5 Covariate collection

To minimize confounding bias, we conducted data analysis by selecting covariates that might influence all-cause mortality in hypertensive patients, based on clinical experience and previous literature (21–23). General demographic information included age, gender, marital status, education, and race. The physical examination encompassed diastolic blood pressure, systolic blood pressure, weight, height, and body mass index (BMI). In addition, we collected a history of a number of lifestyle habits or diseases that may affect hypertension, which included smoking, alcohol consumption, physical activity, use of antihypertensive drugs, diabetes mellitus, cardiovascular disease (CVD), and kidney disease. Further, we collected some laboratory markers that may affect hypertension, which include lymphocytes, neutrophils, serum creatinine, serum uric acid, triglyceride, cholesterol, HDL cholesterol, LDL cholesterol, albumin, and c-reactive protein (CRP). Physical activity status was assessed by the metabolic equivalent of task (MET) and categorized as ideal, moderate, and poor. Based on self-report, smoking history was categorized as present, former, and never. Drinking status is categorized as never, former, mild, moderate or heavy. Cardiovascular disease is defined as having any heart attack, angina, congestive heart failure (CHF), coronary heart disease (CHD), or stroke. Diabetes mellitus was determined by self-reported physician diagnosis, use of glucose-lowering medication or insulin, fasting blood glucose level equal to or greater than 7.0 mmol/L, glycosylated hemoglobin (HbA1c) level greater than 6.5%, random blood glucose level, and two-hour oral glucose tolerance test (OGTT) blood glucose level equal to or greater than 11.1 mmol/L. The estimated glomerular filtration rate (eGFR) was calculated using the CKD Epidemiology Collaboration equation (24). Patients were categorized into two groups based on their eGFR levels: the mild decline group (30–59 ml/min/1.73 m^2^) and the moderate to severe decline group (<30 ml/min/1.73 m^2^).

### 2.6 Evaluation of follow-up results

The study examined two primary outcomes: cardiovascular mortality and all-cause mortality. All-cause mortality was defined as death resulting from any cause. Cardiovascular deaths were identified by referencing the International Statistical Classification of Diseases, 10th Revision (ICD-10) codes I00-I09, I11, I13, and I20-I51. We employed the NHANES public-use linked mortality file as of December 31, 2019. This connection was established through the use of a probabilistic matching algorithm, linking the National Center for Health Statistics (NCHS) with the National Death Index (NDI) (25).

### 2.7 Statistical analysis

In adherence to NHANES Analytical Guidelines, we accounted for complex sampling designs and sample sizes during data analysis (26). Sample weights were calculated to enhance data representativeness. Continuous variables were expressed as weighted mean ± standard deviation, and differences between groups were evaluated using one-way ANOVA. Categorical variables were presented as frequencies and percentages and compared using Rao-Scott's *χ*^2^ test. Kaplan-Meier curves and log-rank tests were employed to assess the survival probability in hypertensive individuals based on PNI levels. The Cox regression model was utilized to calculate hazard ratios (HR) and 95% confidence intervals (CI) in order to investigate the relationship between PNI and the prevalence of all-cause and cardiovascular mortality. The reference category for PNI was the categorical normal group (>49). Three adjustment models were applied: Model 1 adjusted for age, ethnicity, marital status, education, gender, diabetes mellitus, cardiovascular disease, chronic kidney disease, physical activity. Model 2: adjusted for Model 1 + uric acid, triglycerides, HDL, LDL, CRP, neutrophils, creatinine. Model 3: adjusted for Model 2 + medication (beta-blockers, ACEI/ARB, calcium channel blockers, diuretics, statins). To explore the continuous relationship between PNI and all-cause and cardiovascular mortality in hypertensive patients, we applied a Cox-restricted cubic spline model at the 5th, 35th, 65th, and 95th percentiles of PNI. Subgroup analyses were conducted to assess the significance of PNI in different populations. All statistical analyses were carried out using the R (version 4.2.3) and python (version 3.11.4). A two-sided *p*-value of less than 0.05 was considered indicative of a statistically significant difference.

## 3 Results

### 3.1 Participant characteristics according to nutrition risk

In this study, we focused on data from nine consecutive NHANES cycles (1999–2016), surveying a total of 18,189 patients with hypertension who completed an interview and underwent MEC screening in the United States. Based on weighted analyses, the mean age of participants was 56.6 years, with 9,230 males and 8,959 females.84.6% had a high school education or higher. The majority of participants were non-Hispanic whites (47.0%). Participants with lower levels of PNI tended to be older and were more likely to be male, with higher prevalence of CVD, diabetes mellitus, and CKD (*P* < 0.05). Notably, higher body mass index was associated with lower levels of PNI (*P* < 0.05). Regarding blood biochemical factors, participants with lower PNI had higher serum creatinine and CRP levels and lower neutrophil counts (*P* < 0.05). In addition, there was no significant difference in educational level among malnutrition risk groups (*P* > 0.05), while marital status, blood pressure, physical activity, drinking status, smoking status, drug utilization had statistically significant differences (*P* < 0.05). The baseline characteristics of the participants are summarized in Table 1.

**Table 1:** Baseline characteristics of the study population according to the quartiles of the PNI.

Characteristics	Overall	Q1 (<49.0)	Q2 (49.0–52.5)	Q3 (52.5–55.5)	Q4 (>55.5)	*P*-value
	*N* = 18,189	*N* = 4,580	*N* = 5,160	*N* = 4,010	*N* = 4,439	
PNI	52.9 ± 0.1	46.5 ± 0.0	51.1 ± 0.0	54.2 ± 0.0	59.0 ± 0.1	<0.001
General information						
Age, years	56.6 ± 0.2	62.3 ± 0.3	58.2 ± 0.3	55.5 ± 0.4	51.1 ± 0.4	<0.001
BMI, kg/m^2^	30.7 ± 0.1	31.5 ± 0.2	30.8 ± 0.1	30.3 ± 0.1	30.2 ± 0.1	<0.001
Gender						<0.001
Male	9,230 (50.7)	2,504 (58.1)	2,691 (53.3)	1,988 (49.0)	2,047 (44.6)	
Female	8,959 (49.3)	2,076 (41.9)	2,469 (46.7)	2,022 (51.0)	2,392 (55.4)	
Ethnic						<0.001
Non-Hispanic white	8,547 (47)	2,246 (71.0)	2,490 (73.0)	1,839 (71.5)	1,972 (70.7)	
Non-Hispanic black	4,408 (24.2)	1,312 (16.7)	1,231 (12.7)	886 (11.5)	979 (11.3)	
Mexican American	2,758 (15.2)	522 (4.2)	789 (5.3)	678 (6.3)	769 (6.5)	
Other race	2,476 (13.6)	500 (8.0)	650 (9.0)	607 (10.8)	719 (11.4)	
Marital status						<0.001
Cohabitation	10,472 (57.6)	2,428 (58.9)	3,003 (62.7)	2,404 (65.2)	2,637 (63.7)	
Solitary	5,563 (30.6)	1,688 (32.1)	1,591 (26.7)	1,148 (23.7)	1,136 (22.1)	
Never married	2,154 (11.8)	464 (9.1)	566 (10.6)	458 (11.1)	666 (14.2)	
Education levels						0.100
High school or above	15,396 (84.6)	3,874 (91.3)	4,366 (92.1)	3,405 (92.8)	3,751 (92.3)	
Less than High School	2,793 (15.4)	706 (8.7)	794 (7.9)	605 (7.2)	688 (7.7)	
Medical situation						
SBP, mmHg	134.4 ± 0.2	135.5 ± 0.5	134.2 ± 0.4	133.7 ± 0.4	134.1 ± 0.4	0.025
DBP, mmHg	74.0 ± 0.2	70.6 ± 0.3	73.8 ± 0.3	74.4 ± 0.3	76.7 ± 0.3	<0.001
Physical activity						0.001
Poor	7,460 (41)	2,045 (43.5)	2,103 (39.3)	1,567 (38.6)	1,745 (38.8)	
Intermediate	9,192 (50.5)	2,225 (49.4)	2,625 (52.3)	2,083 (52.5)	2,259 (51.5)	
Ideal	1,537 (8.5)	310 (7.1)	432 (8.5)	360 (8.8)	435 (9.8)	
Cardiovascular Disease	3,716 (20.4)	1,301 (25.7)	1,097 (18.4)	686 (14.9)	632 (11.5)	<0.001
Diabetes	5,237 (28.8)	1,504 (28.3)	1,460 (23.5)	1,083 (22.2)	1,190 (20.2)	<0.001
Chronic kidney disease						<0.001
Mild	2,821 (12.97)	1,075 (21.04)	823 (13.74)	517 (11.07)	406 (7.12)	
Moderate to severe	399 (1.47)	220 (3.63)	104 (1.38)	45 (0.70)	30 (0.42)	
Drinking status						<0.001
Never	4,089 (22.5)	1,123 (21.4)	1,170 (18.0)	837 (16.9)	959 (17.6)	
Former	4,282 (23.5)	1,251 (25.1)	1,194 (19.7)	901 (19.3)	936 (18.0)	
Mild	5,466 (30.1)	1,353 (33.2)	1,628 (36.3)	1,265 (35.3)	1,220 (30.1)	
Moderate or heavy	4,352 (23.9)	853 (20.3)	1,168 (26.0)	1,007 (28.5)	1,324 (34.2)	
Smoking status						<0.001
Never	9,175 (50.4)	2,307 (50.3)	2,665 (51.6)	2,037 (49.1)	2,166 (47.2)	
Former	5,674 (31.2)	1,645 (36.1)	1,679 (32.4)	1,206 (31.5)	1,144 (26.3)	
Now	3,340 (18.4)	628 (13.6)	816 (16.0)	767 (19.3)	1,129 (26.6)	
Drug utilization						
Beta-blockers	13,885 (76.3)	3,179 (69.7)	3,897 (75.7)	3,148 (78.5)	3,661 (83.8)	<0.001
CCB	14,487 (79.6)	3,536 (80.2)	4,124 (82.6)	3,213 (82.7)	3,614 (85.2)	<0.001
ACEI/ARB	10,823 (59.5)	2,517 (54.5)	2,997 (58.5)	2,441 (60.7)	2,868 (66.8)	<0.001
Diuretics	12,854 (70.7)	2,949 (65.2)	3,634 (71.1)	2,885 (72.5)	3,386 (78.1)	<0.001
Statins	12,985 (71.4)	3,165 (68.9)	3,634 (70.5)	2,873 (72.2)	3,313 (76.4)	<0.001
Laboratory examinations						
Neutrophil, 10^9 ^/L	2.08 ± 0.01	1.49 ± 0.01	1.84 ± 0.01	2.16 ± 0.01	2.78 ± 0.02	<0.001
Lymphocyte, 10^9 ^/L	4.44 ± 0.02	4.34 ± 0.03	4.24 ± 0.03	4.41 ± 0.04	4.76 ± 0.04	<0.001
Cr, μmol/L	83.63 ± 0.46	92.66 ± 1.31	83.03 ± 0.84	80.75 ± 0.62	79.25 ± 0.42	<0.001
UA, μmol/L	343.83 ± 0.98	341.67 ± 1.82	340.02 ± 1.74	344.59 ± 1.79	348.98 ± 2.00	0.002
TG, mmol/L	2.20 ± 0.02	1.83 ± 0.03	2.06 ± 0.03	2.24 ± 0.04	2.61 ± 0.05	<0.001
TC, mmol/L	5.18 ± 0.01	4.94 ± 0.02	5.12 ± 0.02	5.24 ± 0.02	5.39 ± 0.03	<0.001
HDL-C, mmol/L	1.35 ± 0.00	1.40 ± 0.01	1.37 ± 0.01	1.34 ± 0.01	1.30 ± 0.01	<0.001
LDL-C, mmol/L	2.83 ± 0.01	2.70 ± 0.02	2.81 ± 0.02	2.88 ± 0.02	2.90 ± 0.02	<0.001
Albumin, g/L	42.48 ± 0.05	39.03 ± 0.05	41.91 ± 0.05	43.41 ± 0.05	45.12 ± 0.06	<0.001
CRP, mg/dl	0.47 ± 0.01	0.74 ± 0.02	0.43 ± 0.01	0.39 ± 0.01	0.36 ± 0.01	<0.001

Continuous variables are expressed as weighted mean ± standard deviation and categorical variables are expressed as frequencies (weighted percentages).

PNI, prognostic nutrition index; BMI, body mass index; LDL-C, low-density lipoprotein cholesterol; HDL-C, high-density lipoprotein cholesterol; CRP, C-reactive protein; SBP, systolic blood pressure; DBP, diastolic blood pressure; CCB, calcium channel antagonist; ACEI/ARB, angiotensin-converting enzyme inhibitors/angiotensin II receptor antagonists.

### 3.2 The relationship between PNI and mortality

**Figure 2:**
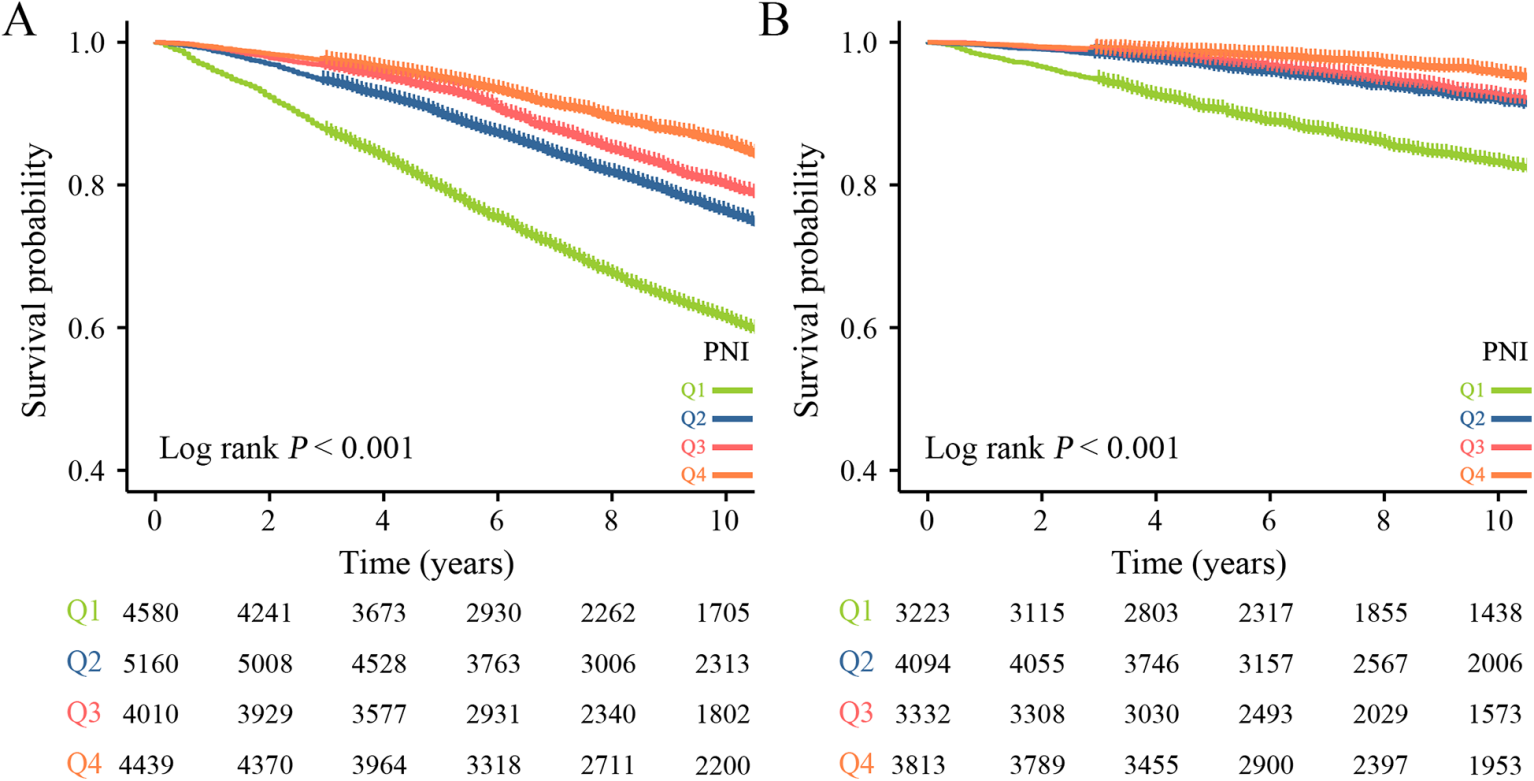
Kaplan–Meier survival curve for mortality across the PNI. **(A)** All-cause mortality. **(B)** Cardiovascular mortality.

**Figure 3:**
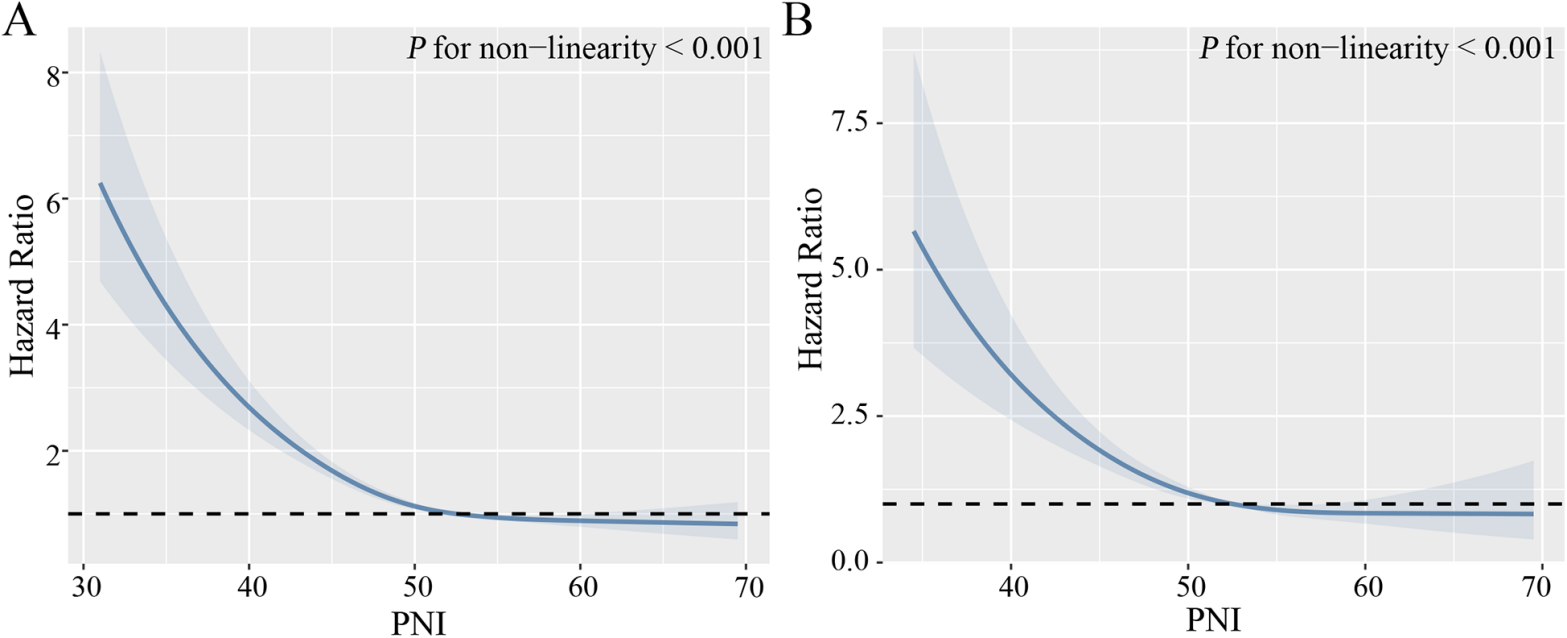
Non-linear relationship between PNI and mortality. **(A)** All-cause mortality. **(B)** Cardiovascular mortality. Adjust for age, gender, ethnicity, marital status, physical activity, smoking, drinking, diabetes mellitus, cardiovascular disease, chronic kidney disease, diastolic blood pressure, systolic blood pressure, serum uric acid, triglycerides, HDL-C, LDL-C, C-reactive protein, and medication (beta-blockers, ACEI/ARB, calcium channel blockers, diuretics, statins).

During a median follow-up time of 103 months, a total of 5,171 (28.43%) hypertensive patients experienced all-cause mortality, 1,444 (7.94%) cardiovascular mortality. Kaplan–Meier curves suggest that hypertensive patients with higher PNI have lower mortality, both all-cause and cardiovascular mortality, in the quartile subgroup of PNI (Figure 2). COX proportional hazard regression analysis showed that PNI was a protective factor for all-cause mortality and cardiovascular mortality, with hazard ratios of 0.906 (95% CI, 0.898–0.913, *P* < 0.001) and 0.878 (95% CI, 0.865–0.892, *P* < 0.001), respectively. Multivariate regression analysis revealed that every one-point increase in PNI was associated with 5%–7% reduction in the risk of all-cause mortality and cardiovascular mortality After adjusting for confounding factors of sex, age, underlying disease, clinical biochemical parameters, and medication history, PNI remained a protective factor for all-cause mortality (HR: 0.953, 95CI: 0.945–0.961, *P* < 0.001) and cardiovascular mortality (HR: 0.928, 95CI: 0.908–0.949, *P* < 0.001). When PNI was assessed by quartile and compared with quartile 1 (<49.0), the hazard ratio for all-cause mortality cardiovascular mortality were <1 for quartile 2 (49.0–52.5), quartile 3 (52.5–55.5) and quartile 4 (>55.5) before and after adjustment for covariates, and the P trend was <0.001 (Table 2). In a fully adjusted restricted cubic spline regression analysis model accounting for potential confounders, we discovered an intriguing L-shaped association between PNI and both all-cause and cardiovascular mortality among hypertensive patients (with all nonlinear *p* values < 0.001). Notably, as PNI values increased, there was a progressive decrease in both all-cause mortality and cardiovascular mortality (Figure 3).

**Table 2:** Cox proportional hazard regression analysis of mortality in participants with PNI and hypertension.

Characteristics	Crude model		Model 1		Model 2		Model 3	
	HR 95% CI	*P*-value	HR 95% CI	*P*-value	HR 95% CI	*P*-value	HR 95% CI	*P*-value
All-cause mortality								
PNI	0.906 (0.898,0.913)	<0.001	0.953 (0.945,0.961)	<0.001	0.958 (0.949,0.966)	<0.001	0.953 (0.945,0.961)	<0.001
Quartiles								
Q1 (<49.0)	Reference		Reference		Reference			
Q2 (49.0–52.5)	0.526 (0.483,0.572)	<0.001	0.691 (0.638,0.750)	<0.001	0.720 (0.663,0.783)	<0.001	0.721 (0.658,0.789)	<0.001
Q3 (52.5–55.5)	0.426 (0.387,0.468)	<0.001	0.677 (0.615,0.744)	<0.001	0.705 (0.640,0.778)	<0.001	0.690 (0.626,0.761)	<0.001
Q4 (>55.5)	0.309 (0.279,0.342)	<0.001	0.594 (0.535,0.659)	<0.001	0.624 (0.560,0.694)	<0.001	0.584 (0.523,0.652)	<0.001
*P* for trend		<0.001		<0.001		<0.001		<0.001
Cardiovascular mortality								
PNI	0.878 (0.865,0.892)	<0.001	0.933 (0.918,0.948)	<0.001	0.924 (0.904,0.944)	<0.001	0.928 (0.908,0.949)	<0.001
Quartiles								
Q1 (<49.0)	Reference		Reference		Reference			
Q2 (49.0–52.5)	0.396 (0.340,0.461)	<0.001	0.557 (0.478,0.650)	<0.001	0.574 (0.493,0.668)	<0.001	0.590 (0.501,0.695)	<0.001
Q3 (52.5–55.5)	0.357 (0.302,0.423)	<0.001	0.626 (0.526,0.747)	<0.001	0.634 (0.533,0.755)	<0.001	0.632 (0.531,0.752)	<0.001
Q4 (>55.5)	0.200 (0.162,0.248)	<0.001	0.438 (0.353,0.543)	<0.001	0.440 (0.356,0.544)	<0.001	0.435 (0.349,0.541)	<0.001
*P* for trend		<0.001		<0.001		<0.001		<0.001

PNI, prognostic nutritional index; HR, hazard ratio; CI, confidence interval.

Crude model: adjusted for none.

Model 1: adjusted for age, ethnicity, marital status, education, gender, diabetes mellitus, cardiovascular disease, chronic kidney disease, physical activity.

Model 2: adjusted for Model 1 + uric acid, triglycerides, HDL, LDL, CRP, neutrophils, creatinine.

Model 3: adjusted for Model 2 + medication (beta-blockers, ACEI/ARB, calcium channel blockers, diuretics, statins).

### 3.3 Subgroup analysis

**Figure 4:**
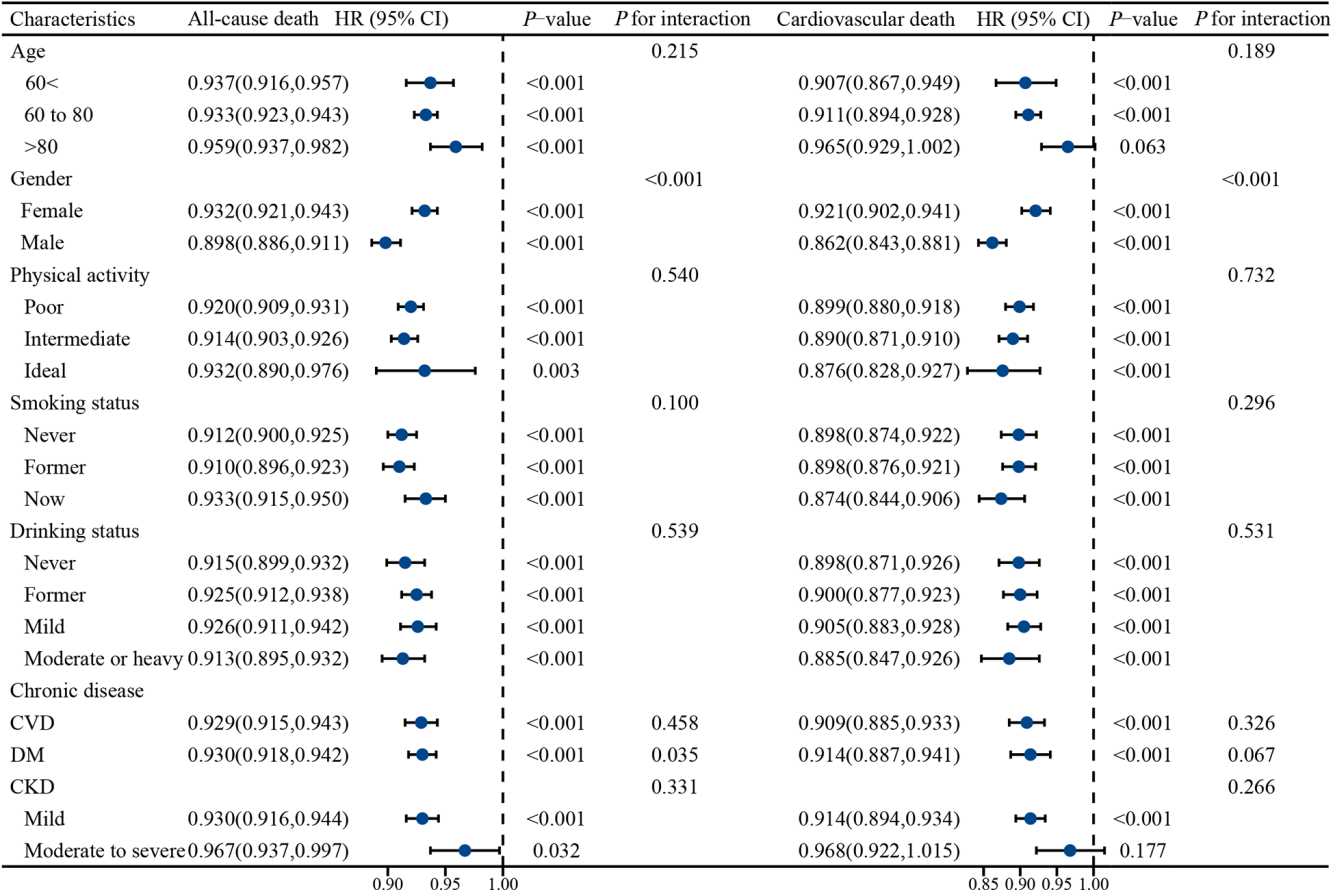
Stratified analysis of the effect of PNI on all-cause mortality and cardiovascular mortality in different populations. Adjusted for Race, marital status, education, consumption and medication (betablockers, ACEI/ARB, calcium channel blockers, diuretics, statins).

This study further analyzed PNI in subgroups of different age groups, gender, physical activity, smoking, alcohol consumption, and chronic diseases (Figure 4**)**. The results showed that PNI was protective against all-cause mortality and cardiovascular death in all subgroups. Notably, we observed an interaction between PNI and both all-cause and cardiovascular deaths in gender (both *P* for interaction <0.001). In addition, no significant association of PNI with cardiovascular mortality was observed in both age >80 years and chronic kidney disease (moderate to severe) (*P* > 0.05).

### 3.4 PNI outperforms other features in predicting all-cause mortality

**Figure 5:**
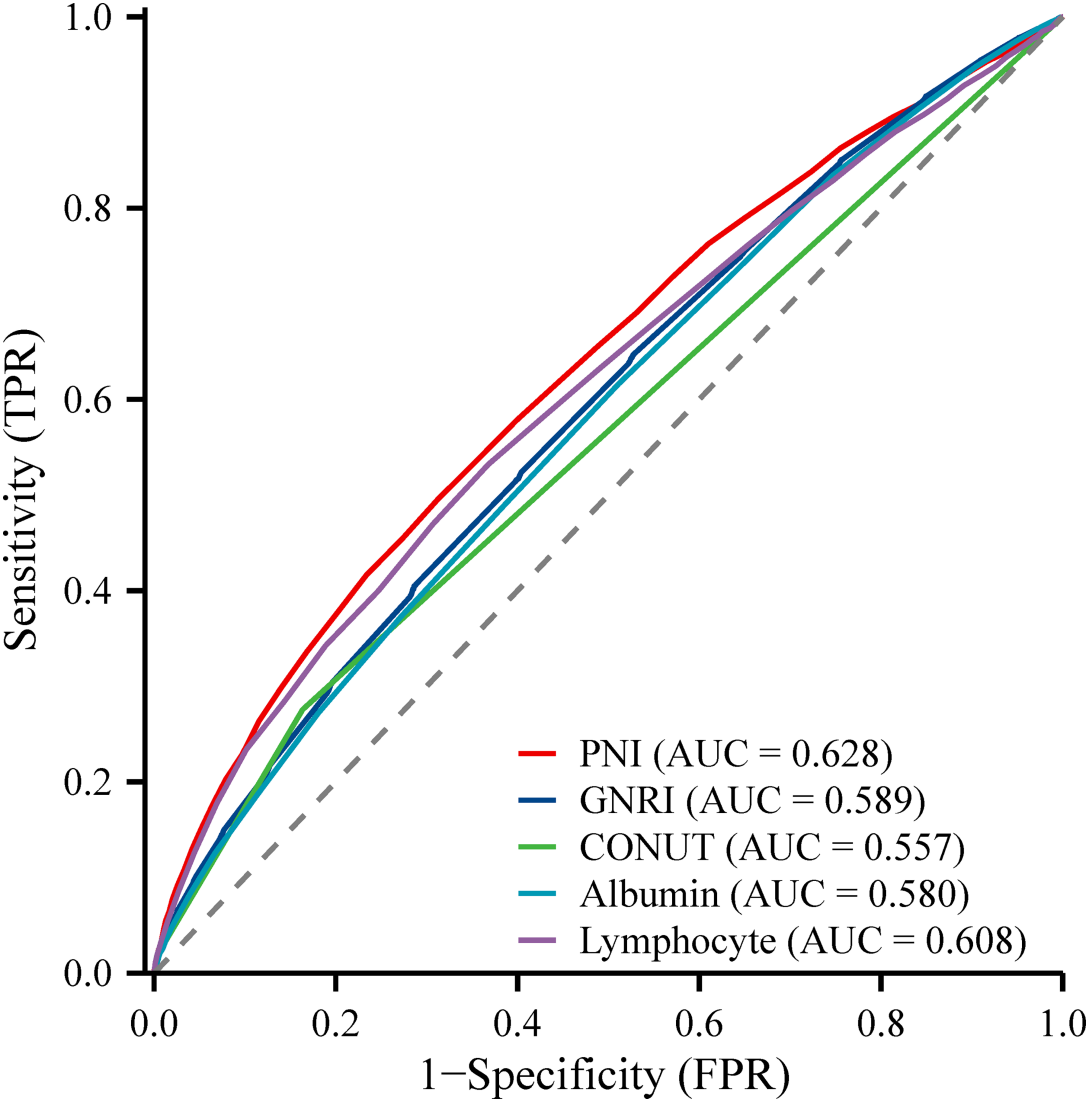
ROC plot of PNI predicting the occurrence of all-cause mortality in participants with hypertension.

In this study, we conducted an assessment of the predictive capabilities of various features for all-cause mortality (Figure 5). Notably, the Prognostic Nutritional Index (PNI) exhibited the highest AUC value, standing at 0.628, and the highest sensitivity, which reached 0.688. These findings unequivocally demonstrate PNI's robust performance in the prediction of all-cause mortality. Its AUC value significantly surpassed that of other features, namely lymphocyte, Albumin, CONUT, and GNRI. Moreover, the heightened sensitivity of PNI further underscores its exceptional ability to accurately identify instances of all-cause mortality (Table 3**)**.

**Table 3:** Analysis of the efficacy of the PNI in predicting all-cause mortality.

Characteristics	AUC	Sensitivity	Specificity	Yoden's index	Cut-off value
Lymphocyte	0.608	0.632	0.532	0.164	1.9
Albumin	0.580	0.489	0.616	0.105	43
CONUT	0.557	0.276	0.837	0.112	1.0
GNRI	0.589	0.596	0.523	0.12	104
PNI	0.628	0.688	0.495	0.183	51

PNI, prognostic nutritional index; GNRI, geriatric nutritional risk index; CONUT, controlled nutritional status; AUC, area under the curve.

## 4 Discussions

The main finding of this study was that lower levels of PNI were associated with increased all-cause and cardiovascular mortality in hypertensive patients. Meanwhile, PNI demonstrated superior predictive power compared to other nutritional indices.

Hypertension is a major modifiable risk factor for cardiovascular and cerebrovascular diseases. Identifying alterable factors is crucial for delaying or preventing hypertension-related target organ damage (27). Several comprehensive nutritional assessment methods have been reported, including Subjective Global Assessment (SGA), Mini Nutritional Assessment (MNA), Malnutrition Inflammation Score (MIS), Geriatric Nutritional Risk Index (GNRI), Controlling Nutritional Status (CONUT) score, and PNI (28). SGA, MNA, and MIS are assessed by experienced clinicians based on patients’ symptoms and physical examinations, which may introduce subjective bias. In contrast, objective nutritional indices such as GNRI, CONUT score, and PNI utilize widely available and cost-effective biomarkers for calculation. PNI includes serum albumin concentration and lymphocyte count, provides a comprehensive evaluation of nutritional, immune, and inflammatory status. The results of this study demonstrated that hypertensive patients with higher PNI levels had lower mortality rates, including both all-cause and cardiovascular mortality, across PNI quartile subgroups. These findings align with previous studies conducted in other populations. For example, lower PNI levels have been reported as independent predictors of short-term adverse outcomes in patients with severe decompensated acute heart failure (29). Cheng et al. also highlighted that PNI was negatively associated with long-term survival in patients with acute heart failure (16). Moreover, PNI has been shown to predict early mortality and complications in cardiac surgery patients (30).

The progression of hypertension is closely linked to mechanisms involving chronic inflammation and immune status. Albumin is a classic marker for assessing malnutrition; however, its concentration is influenced not only by protein intake but also by factors such as overhydration, inflammation, or other physiological disturbances (31). Studies have demonstrated that when serum albumin levels drop below 3.5 g/dl, the risk of mortality quadruples compared to individuals with higher levels, and levels below 3 g/dl indicate a critical condition (32). Nutritional status reflects a patient's overall health and protein reserves, and a decrease in PNI may indicate poor general condition and reduced protein stores. Reduced lymphocyte levels often indicate immunosuppression or heightened inflammatory responses (33). In chronic disease and malnourished states, impaired immune capacity may lead to increased risks of infection, uncontrolled inflammation, and organ damage. This study found that participants with lower PNI levels had higher serum CRP levels. Increasing evidence suggests that prolonged hyperactivation of immune cells and subsequent low-grade inflammation are significant drivers of hypertension and its fatal complications (34, 35). These findings further validate PNI's representation of the ongoing inflammatory activation process.

The results of this study demonstrate that PNI effectively predicts cardiovascular and all-cause mortality across various subgroups, highlighting its broad applicability. Furthermore, this study identified a significant sex-based interaction between PNI and mortality. Elevated PNI levels were more strongly associated with lower mortality in men than in women. This could be attributed to men's heightened sensitivity to malnutrition and immune function decline, combined with behavioral patterns and metabolic characteristics that exacerbate their vulnerability (36). Conversely, women may benefit from physiological protective mechanisms, such as higher estrogen levels, which could mitigate the adverse effects of nutrition status to some extent. Additionally, men's tendencies toward neglecting nutritional management might contribute to their higher PNI-related risks (37).

In this study, PNI exhibited a significantly higher AUC value and sensitivity compared to other nutritional indices, indicating its superior performance in assessing mortality risk among hypertensive patients. GNRI incorporates body mass index (BMI) and albumin levels, tends to overestimate nutritional status in obese individuals (38). Similarly, this study revealed that participants with lower PNI levels often had higher BMI values. The World Health Organization (WHO) has highlighted the “double burden of malnutrition,” where both malnutrition and overweight coexist, posing a serious and growing global health challenge (39). The CONUT score, which includes albumin, lymphocyte count, and total cholesterol, has demonstrated good predictive performance in certain populations. However, some patients with chronic diseases often require long-term lipid-lowering therapies, such as statins, to reduce cardiovascular risk. These medications can lead to decreased cholesterol levels, potentially compromising the accuracy of CONUT in this population (20). Although PNI has relatively lower specificity, its superior sensitivity and AUC value make it an ideal tool for screening mortality risk in hypertensive patients. High sensitivity ensures that more high-risk patients are accurately identified, which is crucial in clinical practice for implementing timely interventions. The issue of lower specificity can be addressed by optimizing application strategies, such as combining PNI with other biomarkers or adjusting its threshold in specific populations.

## 5 Advantages and limitations

Our study has several advantages. First, our study is the first to show an association between PNI levels and mortality in a longitudinal cohort study of a large number of persons with hypertension. Second, we explored the relationship between PNI and cardiovascular mortality and all-cause mortality, respectively. In addition, we adjusted for as many confounding factors as possible, so the results may be more convincing. There are also several limitations to this study. First and foremost, despite our rigorous adjustment for baseline clinical characteristics, our observations may be influenced by unmeasured and unknown confounders. Furthermore, because the NHANES study collected data at one point in time, nutritional data such as serum albumin, height, and weight were recorded only once for all participants, which may lead to bias in PNI calculations.

## 6 Conclusions

This study confirms that lower PNI scores are highly associated with the risk of all-cause mortality and cardiovascular mortality in persons with hypertension. To avoid premature death among adults with hypertension in the United States, it is recommended that they focus on a balanced nutritional intake in their daily lives. Clinical care workers should also pay attention to assessing the nutritional status of patients and give them timely and appropriate dietary guidance. This study provides a significant reference for reducing premature mortality in the hypertension population with adequate nutritional intake as a primary prevention strategy!

## Data Availability

The datasets presented in this study can be found in online repositories. The names of the repository/repositories and accession number(s) can be found below: this study is a secondary exploration of the NHANES public database. The data used in the manuscript can be accessed and downloaded from the website https://wwwn.cdc.gov/nchs/nhanes/search/default.aspx.
